# 3D laparoscopic surgery for the treatment of adult Morgagni hernia: a case report

**DOI:** 10.1186/s13019-025-03728-9

**Published:** 2025-12-29

**Authors:** Jian-xian Zhang, Hua-bin Cheng, Shu-liang Li, Yan Xue

**Affiliations:** 1 Department of Gastrointestinal Surgery, The Second People’s Hospital of Liaocheng, Linqing, 252600 Shandong China; 2 Department of Gastroenterology, The Second People’s Hospital of Liaocheng, Linqing, 252600 Shandong China

**Keywords:** Laparoscopic surgery, Congenital diaphragmatic hernia, Treatment

## Abstract

This case report describes the successful treatment of a 64-year-old female patient with Morgagni hernia, a rare type of diaphragmatic hernia, using 3D laparoscopic surgery. The patient presented with a history of chest tightness and shortness of breath for over 10 years, which worsened over the past year. The patient underwent a 3D laparoscopic right retrosternal hernia tension-free repair (IPOM) combined with laparoscopic adhesion lysis on April 15, 2024. The patient recovered smoothly after the operation.

Morgagni hernia, also known as congenital retrosternal hernia or parastomal hernia, is a special type of diaphragmatic hernia (congenital diaphragmatic hernia, CDH), accounting for about 3–5% of all congenital diaphragmatic hernias, with a low incidence rate. Depending on the location, a defect in the right diaphragm is called Morgagni hernia, while a defect in the left diaphragm is called Larrey hernia, and it is rare for both sides to be involved [1]. Morgagni hernia defects generally have no clinical symptoms and are mostly discovered in older adults or adults due to respiratory or digestive tract symptoms. Surgical repair of the diaphragmatic defect is the only way to treat Morgagni hernia, and in recent years, there has been an increase in reports of laparoscopic surgery. Our unit treated an adult patient with retrosternal hernia, and a 3D laparoscopic retrosternal hernia repair was performed, with a good recovery. The report is as follows.

## Case introduction

**Fig. 1:**
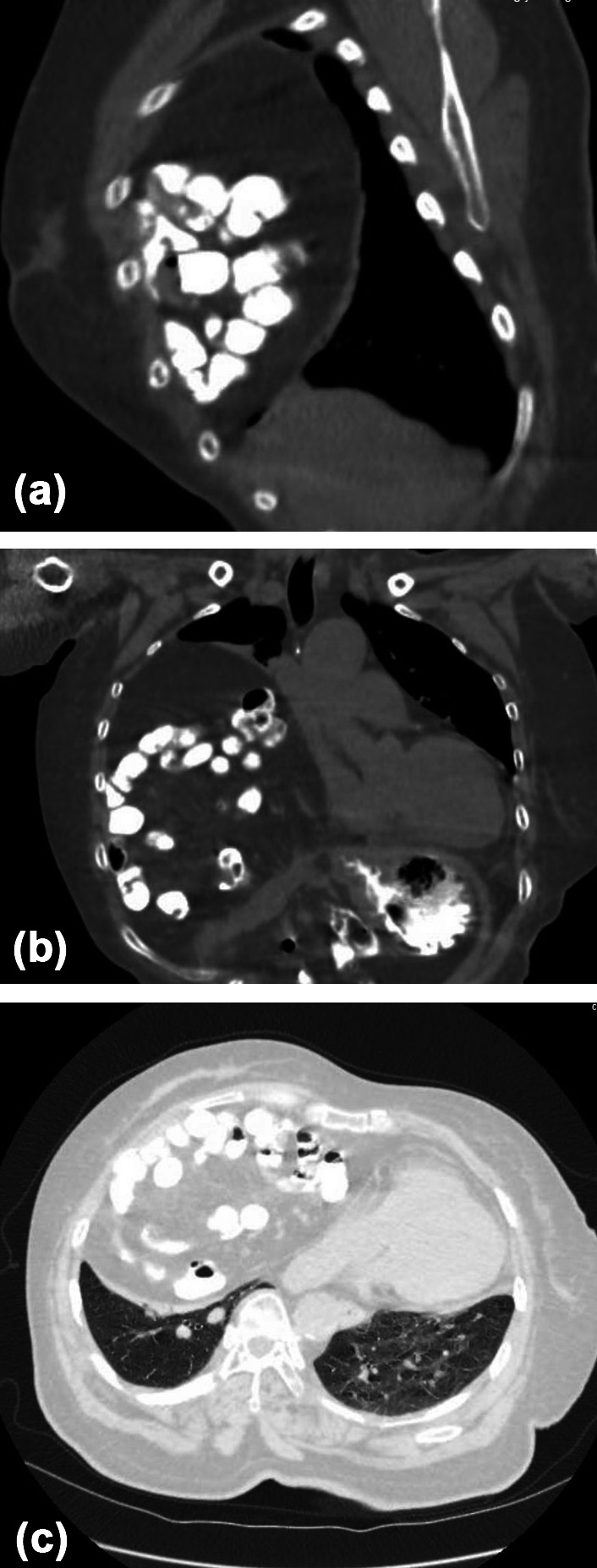
The hernia contents have entered the thoracic cavity.(a)Sagittal CT image.(b)Coronal CT image.(c)Axial CT image.

The patient is a 64-year-old female. She was admitted on April 10, 2024, with a chief complaint of “chest tightness and shortness of breath for more than 10 years, worsening for more than a year.” The patient had no obvious cause for chest tightness and shortness of breath more than 10 years ago, which worsened with activity and was not taken seriously. More than a year ago, the patient’s chest tightness and shortness of breath worsened, accompanied by cough and sputum. She came to our outpatient clinic for further treatment, she was admitted to the hospital with “right thoracic hernia.” She has a history of “coronary heart disease, cerebral infarction” for more than 10 years and has been taking “aspirin, simvastatin, and Danshen pills” for treatment. After admission, a chest CT plain scan showed: consistent with right thoracic hernia (retrosternal hernia), with partial atelectasis of the right lung. X-ray film: (1) Gastritis signs; (2) Consistent with diaphragmatic hernia. Gastroscopy showed: mild esophageal hiatal hernia, erosive gastritis. See Fig. 1a–c.

**Fig. 2:**
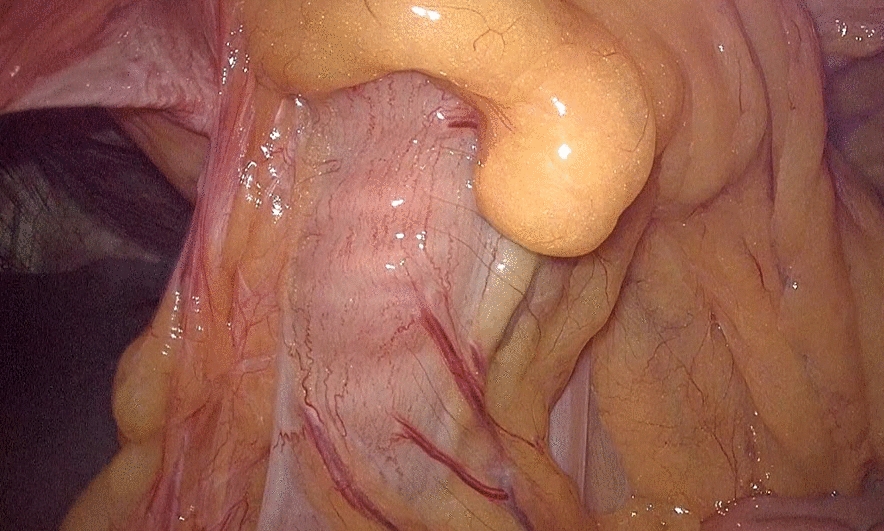
The transverse colon, greater omentum, and part of the small intestine have herniated into the thoracic cavity

**Fig. 3:**
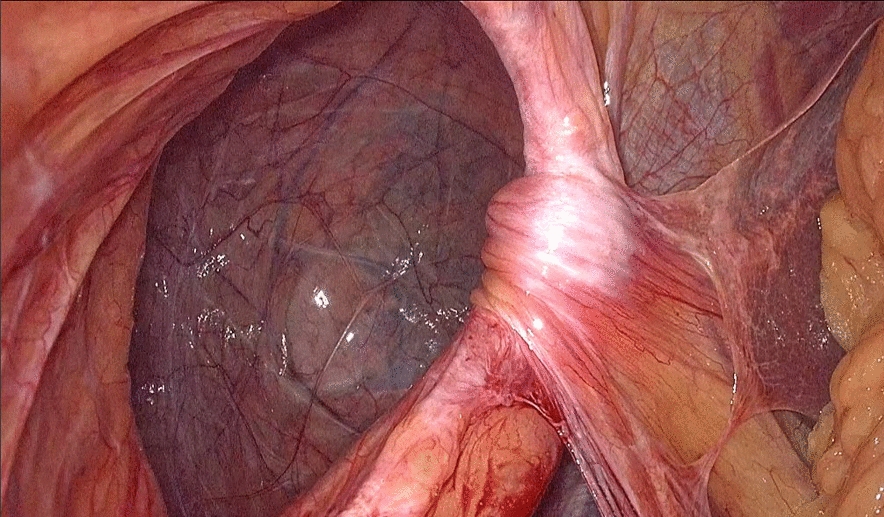
After reducing the hernia contents, a large hernia sac is visible

**Fig. 4:**
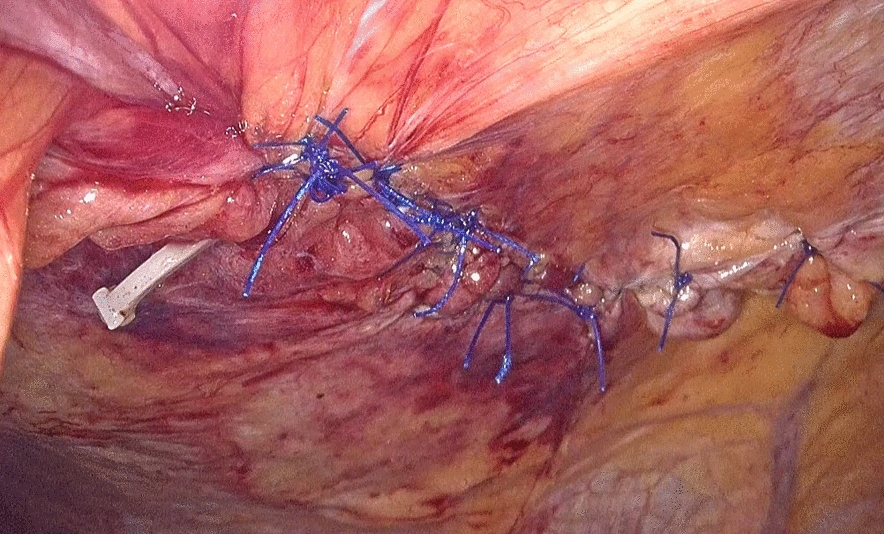
Interrupted sutures are placed in the hernia sac

**Fig. 5:**
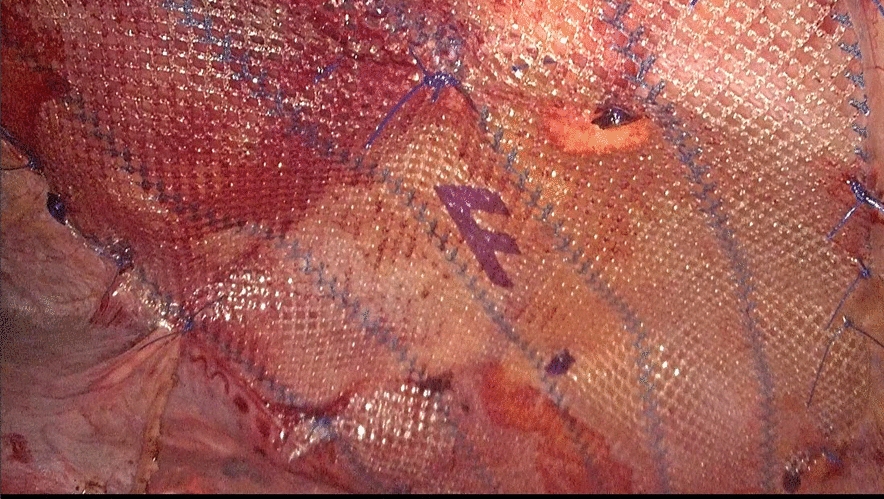
An anti-adhesion patch is placed

Preoperative diagnosis: 1. Right retrosternal hernia (thoracic hernia) 2. Coronary atherosclerotic heart disease 3. Bilateral pulmonary nodules 4. Right lung atelectasis 5. Gallbladder stones 6. Cerebral infarction 7. Esophageal hiatal hernia 8. Right lung nodule 9. Erosive gastritis 10. Kidney stones in both kidneys. After assessment, there were no contraindications to surgery, and on April 15, 2024, under general anesthesia, 3D laparoscopic right retrosternal hernia tension-free repair (IPOM) + laparoscopic adhesion lysis was performed. Surgical method: After successful anesthesia, the patient was placed in a supine position, the surgical field skin was routinely disinfected, and sterile surgical sheets were laid. A small incision was made at the upper edge of the navel, a pneumoperitoneum needle was inserted, CO_2_ was filled, and the pneumoperitoneum pressure was set to 12 mmHg. A 5 mm trocar was placed at 5 cm above the navel on the right axillary midline, and a 12 mm trocar was placed at the level of the navel on the left axillary midline, and laparoscopic operating instruments were inserted. The position was adjusted with the head high and the feet low, and the hernia content was found to be the greater omentum, part of the small intestine, and the transverse colon. The hernia content was adhered to the abdominal wall, carefully separated, and the hernia content was returned to the abdominal cavity. The hernia sac was located behind the sternum, and the hernia ring diameter was about 10 cm. First, the hernia ring was closed with 3–0 barbed thread, then reinforced with interrupted sutures with Prolene thread, and the anti-adhesion patch was fixed to the weak part of the diaphragm with a fixator and Prolene thread. The abdominal cavity was checked for no bleeding, and the surgery ended. Each trocar was removed, and the trocar hole was checked for no bleeding. Each incision was closed with absorbable sutures. Intraoperative bleeding was 10 mL, and the surgery was smooth. See Figs. 2, 3, 4, and 5.

## Discussion

In 1760, Morgagni hernia was first discovered and reported by Morgagni during the dissection of a corpse. The formation of Morgagni hernia is due to congenital developmental disorders of the diaphragm; the abdominal cavity organs protrude into the thoracic cavity or mediastinum through the area of the anterior medial part of the diaphragm below the sternum (Morgagni foramen), also known as Morgagni foramen hernia. Generally, left diaphragmatic hernia is less common, which is considered to be related to the protective effect of the pericardial diaphragm on the left anterior part of the diaphragm. Abdominal cavity organs with mesentery have the potential to hernia into the costal triangle gap, and the most easily herniated abdominal organs are the colon, omentum, stomach, and small intestine, and there is also a possibility of liver herniation [2]. Most patients with Morgagni hernia may have no obvious symptoms and lack typical clinical characteristics, which can easily lead to misdiagnosis. This patient was misdiagnosed with “heart disease” and underwent treatment for a period of time. When the patient’s lung is compressed by the hernia content, there may be symptoms such as cough, difficulty breathing, or recurrent lung infections [3]; when the hernia content is incarcerated, there may also be symptoms such as nausea, vomiting, and abdominal pain. The diagnosis of Morgagni hernia mainly relies on imaging examinations; the typical sign on the chest anteroposterior X-ray film is a sharp-edged circular shadow protruding into the lung field from one side of the cardiophrenic angle. CT examination can clearly display the location, shape, size, and content of the hernia sac, the relationship between the hernia sac and the thoracic and abdominal cavities, the diaphragm, and adjacent organs, which can usually diagnose the disease [4]. The hernia sac of this case of Morgagni hernia is huge, and it is very rare to be reported in previous literature. Due to the risk of internal hernia and incarceration, Morgagni should be treated as soon as the diagnosis is confirmed, even if there are no symptoms [5]. The surgical repair of the diaphragmatic defect is divided into thoracic or abdominal surgery [6]. For elderly patients with poor cardiopulmonary function and symptoms, abdominal surgery is the first choice because abdominal surgery can avoid interference with cardiopulmonary function and circulation. With the increasing maturity of laparoscopic technology, surgery is now mostly completed under laparoscopy [7]. Laparoscopic surgery has the advantages of less injury, less pain, and faster recovery. For smaller hernia rings, it is generally only necessary to use non-absorbable sutures for interrupted suture of the defect. For hernia rings larger than 5 cm, patch repair can be considered to reduce the risk of recurrence. In this single case, a 10-cm Morgagni defect was successfully repaired via a 3-dimensional laparoscopic approach with a prosthetic mesh. The patient was discharged without immediate complications, and the short-term (6-month) follow-up has not shown clinical or radiological recurrence. Some literature reports that the recurrence rate of diaphragmatic hernia after minimally invasive treatment is 5.0–23.1% [8, 9], and the details of surgical operation are very important for the recovery after diaphragmatic hernia surgery; the diaphragmatic defect is often poorly developed, especially the front edge lacks tough fascia tissue, and there is a risk of local tearing after suture, leading to the risk of recurrence [10]. In this case, we used advanced 3D laparoscopy for surgery, which enhanced the surgeon’s perception of the subject, depth, and level, restored the real three-dimensional surgical vision and precise spatial positioning. However, this report is limited to one patient; therefore, no statement can be made about the superiority of 3D laparoscopy over conventional 2D laparoscopy or open repair. There is a controversy about whether to remove the hernia sac during surgery for Morgagni hernia. Removing the hernia sac increases the difficulty of the operation and the risk of injury to the pericardium, pleura, and diaphragmatic nerves. Retaining the hernia sac and directly suturing the diaphragmatic defect reduces the risk of secondary injury caused by the removal of the hernia sac. Taha et al. [11] reported that non-resection of the hernia sac surgery is safe. Although we elected to leave the hernia sac intact and observed no early adverse events, the effect of sac preservation on long-term recurrence, mesh integration, or chronic pain remains unknown. The literature quotes recurrence rates of 5–23% after minimally invasive repair; our 6-month follow-up is too short to alter that range. Diaphragmatic tissue at the anterior rim is often fragile; whether the 10-cm defect in this well-compensated (ASA II) patient will remain intact over years cannot be predicted from this report. Finally, 3D laparoscopic systems are not universally available and their cost-effectiveness was not examined. Multicentre registries or controlled trials with systematic long-term imaging are required before any recommendation regarding routine use of 3D optics, mesh size, or sac management can be made.

## Data Availability

No datasets were generated or analysed during the current study.
